# Fluorescence-Based Detection of *KRAS* Mutations in Genomic DNA Using Magnetic Bead-Coupled LDR Assay

**DOI:** 10.3390/mps8060142

**Published:** 2025-12-01

**Authors:** Chika Morimoto, Masahiko Hashimoto

**Affiliations:** Department of Chemical Engineering and Materials Science, Faculty of Science and Engineering, Doshisha University, 1-3 Tataramiyakodani, Kyotanabe 610-0321, Japan

**Keywords:** fluorescence detection, ligase detection reaction (LDR), magnetic beads, polymerase chain reaction, single-nucleotide variations (SNVs)

## Abstract

We previously developed a bead-coupled ligase detection reaction (LDR) assay that enables simple and rapid detection of single-nucleotide variations (SNVs) using synthetic oligonucleotide templates. In the present study, this approach was extended to genomic DNA extracted from colorectal cancer cell lines to evaluate its applicability to clinically relevant samples. Targeting codon 12 of the *KRAS* gene, PCR-amplified products served as templates for bead-coupled LDR, and fluorescence excitation–emission matrix (EEM) analysis was employed for signal readout. The four fluorophores used in the assay exhibited distinct spectral properties, allowing their signals to be clearly resolved within the EEM profiles. This mapping provided characteristic fluorescence signatures that revealed the underlying genotypes, enabling not only the distinction between homozygous and heterozygous states but also the precise identification of allele compositions, as exemplified by G/A, T/T, G/G, and G/C in colorectal cancer cell lines. The single-tube workflow, integrating magnetic bead capture with fluorescence-based detection, demonstrated robustness, speed, and cost-effectiveness compared with conventional mutation detection methods. These findings confirm that the LDR–EEM platform can be successfully applied to genomic DNA analysis, underscoring its potential as an accessible and reliable tool for SNV detection in both research and diagnostic contexts.

## 1. Introduction

Single-nucleotide variations (SNVs), encompassing both single-nucleotide polymorphisms (SNPs) and point mutations, are among the most common types of genetic alterations across genomes and play significant roles in disease susceptibility, genetic diversity, and drug response [1,2]. Many point mutations are associated with various diseases, including cancer, highlighting the need for rapid and reliable detection techniques in both research and clinical diagnostics [3,4,5]. Advanced technologies such as next-generation sequencing and DNA microarrays enable precise and comprehensive analysis of genetic variations [6,7,8]. However, their complexity and cost often limit their utility, particularly in routine and point-of-care applications.

The ligase detection reaction (LDR), first introduced by Barany in 1991, is a practical and versatile method for the highly specific identification of nucleotide substitutions at defined positions in a DNA sequence [9]. In LDR, ligation occurs between a discriminating primer and a common primer only when the 3′-terminal base of the discriminating primer perfectly matches the target nucleotide in the DNA template, thereby ensuring precise single-base discrimination. Since that pioneering study, numerous ligase-based mutation-detection strategies have been developed. Early implementations enabled identification of clinically relevant point mutations in oncogenes such as *KRAS* codon 12 variants [10,11] and drug-resistance mutations in viral and bacterial pathogens [12,13]. In the early stages, electrophoresis was commonly used to separate and detect ligated products [10,11,14,15,16,17,18,19,20], which often made the procedure time-consuming and labor-intensive. Alternatively, microarray-based methods were introduced to enable parallel analysis of multiple targets [21,22,23,24,25], but these approaches typically required additional hybridization and washing steps that increased procedural complexity. Detection schemes then diversified, including flow-cytometric [26] and suspension-array-based [27] approaches, as well as microfluidic chip-integrated [28] and optical or nanomaterial-assisted readouts such as surface-enhanced Raman scattering [29]. Collectively, these developments underscored both the remarkable specificity of LDR and the need for simpler, more accessible detection formats beyond electrophoretic, array-based, or other instrument-intensive readouts.

Building upon this foundation, our previous study developed a single-tube LDR assay that combined fluorescently labeled allele-specific primers with solid-phase capture on magnetic beads, thereby enabling selective detection without electrophoresis or microarrays [30]. This workflow simplified sample handling and reduced processing time while maintaining high specificity, offering a practical route for rapid SNV analysis in laboratory and clinical settings. Importantly, the use of a standard spectrofluorometer, rather than specialized array or flow-cytometric instrumentation, underscores the method’s accessibility for typical molecular biology laboratories. However, the effectiveness of this assay was previously demonstrated only with synthetic oligonucleotide templates, and its applicability to genomic DNA extracted from biological samples remained to be verified.

In the present study, we focus on extending this established assay platform to genomic DNA extracted from colorectal cancer cell lines to evaluate its applicability to clinically relevant samples, particularly *KRAS* codon 12 mutations that are associated with tumor progression, treatment resistance, and poor prognosis [31]. Furthermore, we integrate the bead-coupled LDR with excitation–emission-matrix (EEM) fluorescence mapping as a direct optical readout, achieving single-tube, fluorophore-encoded multiplex genotyping of PCR-amplified genomic DNA using standard spectrofluorometric instrumentation. This configuration eliminates the need for electrophoretic or array-based separation steps, providing a streamlined and versatile platform for mutation analysis.

## 2. Materials and Methods

### 2.1. Materials

All reagents used in this study were purchased from commercial sources and were of analytical-grade. Oligonucleotides, including PCR primers, allele-specific discriminating primers, each labeled with a different fluorophore (HEX™, TAMRA™ (TMR), ROX™, or Alexa Fluor^®^ 647 (AF647)), a biotinylated common primer, and synthetic template DNA, were obtained from Integrated DNA Technologies (Coralville, IA, USA). The sequences of all oligonucleotides used in this study for PCR and LDR are provided in Table 1. The genomic DNA templates were extracted from LS180, SW620, HT29, and SW1116 cell lines (ATCC), which represent colorectal cancer models with different *KRAS* codon 12 genotypes. These cell lines were selected to include both wild-type and various mutant alleles at codon 12, as summarized in Table 2. This diversity in mutation status provides a robust framework for evaluating the ability of the assay to detect clinically significant *KRAS* mutations. PCR reagents, including dNTPs, *Taq* DNA Polymerase, ThermoPol reaction buffer, and LDR-specific reagents, such as the specialized LDR buffer and *Taq* DNA ligase, were supplied by New England Biolabs Japan Inc. (Tokyo, Japan). Streptavidin-coated magnetic beads (Dynabeads^TM^ M-270) were obtained from Thermo Fisher Scientific K.K. (Tokyo, Japan).

### 2.2. Preparation of Template DNA and PCR

**Figure 1:**
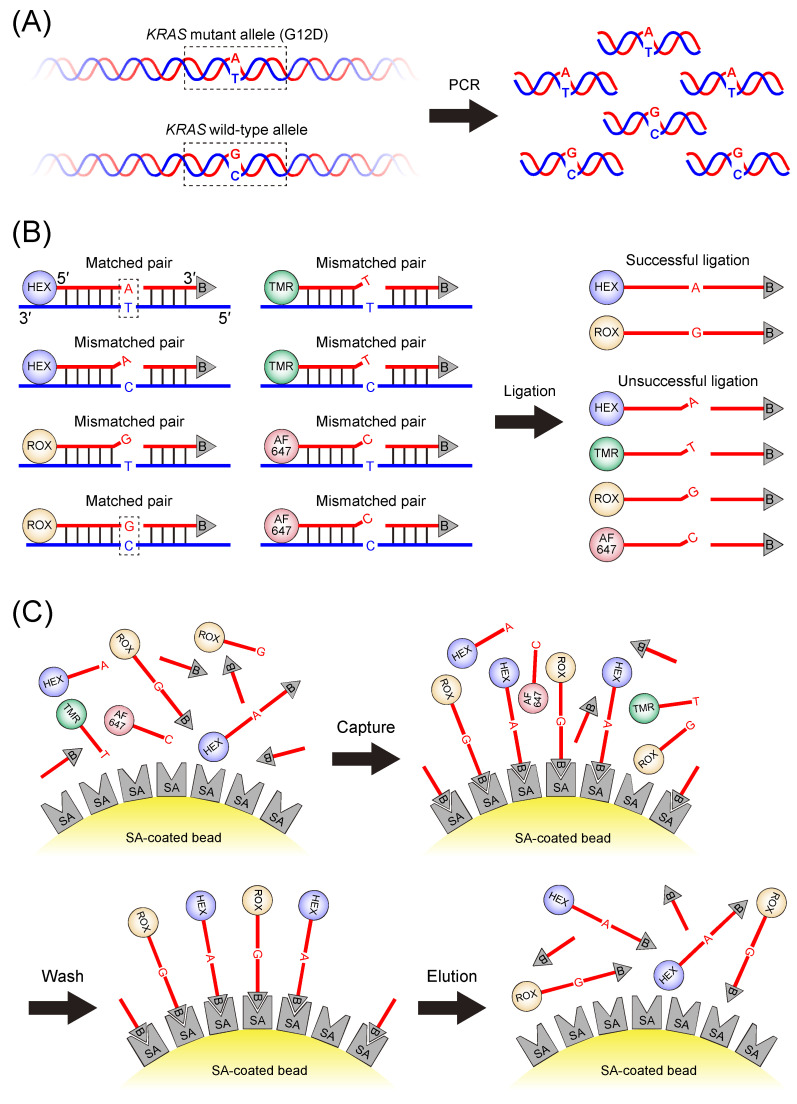
Workflow of the single-tube ligase detection reaction (LDR)-based assay for point mutation analysis. (**A**) PCR amplification of a region of interest. Genomic DNA extracted from cell lines was used as a template to amplify a fragment containing the target site. The figure illustrates a heterozygous G12D (G/A) mutation in *KRAS* codon 12 as a representative example. (**B**) LDR. A set of four fluorescently labeled discriminating primers and a biotinylated common primer are annealed to the template DNA. Ligation occurs selectively when the 3′-terminal base of a discriminating primer perfectly matches the complementary base in the template DNA, generating LDR products. As illustrated for G12D heterozygous alleles in this figure, the LDR produced ROX-labeled LDR products for wild-type sequences and HEX-labeled LDR products for mutant sequences. These products can subsequently be distinguished through fluorescence spectral analysis. (**C**) Selective recovery of LDR products. Ligation products were captured on streptavidin-coated magnetic beads, and unreacted primers were removed through washing steps. The purified LDR products were eluted from the bead surface for fluorescence spectral analysis. Here, HEX™; ROX™; TMR (TAMRA™); and AF647 (Alexa Fluor^®^ 647) are fluorophores with distinct spectral properties. B represents biotin, and SA represents streptavidin.

The genomic DNA was extracted from the cell lines using the Wizard^®^ SV Genomic DNA Purification System (Promega, Madison, WI, USA) according to the manufacturer’s protocol. The genomic DNA extracted from the cell lines served as template DNA for amplifying the exon 2 region of the *KRAS* gene, including codon 12 mutations, by PCR to generate a 290 bp amplicon (Figure 1A). Each 50 µL PCR reaction mixture contained the following final concentrations: 1× ThermoPol buffer, 300 µM dNTPs, 0.5 µM forward primer, 0.5 µM reverse primer, 0.025 U/µL *Taq* DNA Polymerase, and template DNA in the range of 10–50 ng, with nuclease-free water added to achieve a total volume of 50 µL. PCR was conducted on a thermal cycler under the following conditions: initial denaturation at 94 °C for 2 min, followed by 35 cycles of denaturation at 94 °C for 15 s, annealing at 61 °C for 30 s, and extension at 72 °C for 18 s, with a final extension at 72 °C for 1 min.

### 2.3. LDR

LDR was performed using the PCR products as template DNA. The reaction mixture (50 µL total) contained the following final concentrations: 1× LDR buffer with additional KCl (final potassium ion concentration of 100 mM), 0.05 µM of each of the four fluorophore-labeled discriminating primers, 0.2 µM biotinylated common primer, 10 nM PCR product, and 0.4 U/µL *Taq* DNA ligase, with nuclease-free water added to achieve a total volume of 50 µL. The LDR buffer was specifically formulated to enhance the activity of *Taq* DNA ligase. Thermal cycling was carried out with an initial denaturation at 94 °C for 2 min, followed by 20 cycles of 94 °C for 30 s and 65 °C for 2 min, resulting in the generation of LDR products. These are ligation products formed by the joining of a fluorophore-labeled discriminating primer and a biotinylated common primer. Ligation occurs specifically at the junction between the 3′-end of the discriminating primer and the 5′-end of the common primer only when the 3′-terminal nucleotide of the discriminating primer is perfectly complementary to the corresponding target base in the template DNA (i.e., the PCR product). This specificity is conferred by *Taq* DNA ligase, which catalyzes phosphodiester bond formation only when full complementarity is present at the ligation junction. The workflow of the LDR process is illustrated in Figure 1B.

### 2.4. Purification of LDR Products

The streptavidin-coated magnetic beads were used to selectively capture the biotin-labeled LDR products. Magnetic beads are advantageous for their ability to aggregate rapidly in a tube using a magnet, enabling efficient separation of solid (i.e., beads) and liquid phases. This simplifies washing steps, allows for easy buffer exchange, and facilitates the removal of residual unbound components after target molecules are captured on the bead surfaces. Ten microliters of stock bead suspension was washed twice with 100 µL of 10× SSC buffer and resuspended in 50 µL of 10× SSC buffer. The LDR mixture (50 µL, after the thermal cycling) was then combined with the 50 µL bead suspension, followed by an incubation at 25 °C for 5 min with gentle rotation to facilitate the immobilization of biotinylated LDR products on the bead surfaces via the biotin-streptavidin interactions. Unbound components were removed by washing the beads three times with 100 µL of 10× SSC buffer. The LDR products were eluted in 50 µL of 98% formamide containing 10 mM EDTA at 75 °C for 5 min to disrupt the biotin-streptavidin interactions. This method was adapted from the previous study [32], which successfully demonstrated this approach for eluting biotin-labeled oligonucleotides. The magnetic beads were then separated using a magnet, and the supernatant containing the eluted LDR products was collected and transferred to a microcuvette for fluorescence spectral measurement. The selective recovery workflow is shown in Figure 1C.

It should be noted that, after the three washing steps, the EEM spectra obtained from the eluates displayed only the expected fluorophore peaks, indicating that unligated fluorescently labeled discriminating primers were effectively removed during bead purification. Unligated biotinylated common primers were co-captured and eluted together with the ligated products but did not contribute to the fluorescence signals because they carry no fluorophore. Considering the manufacturer-reported binding capacity (~200 pmol per mg bead for Dynabeads M-270) and the very high streptavidin–biotin affinity (*K*_A_ ≈ 2 × 10^13^ M^−1^ [33]), the capture step is not expected to be capacity-limited and is consistent with efficient recovery under the present conditions.

### 2.5. Fluorescence Measurements

Fluorescence spectra of the purified LDR products were recorded using a spectrofluorometer (FP-6500, JASCO, Tokyo, Japan). For each measurement, the emission spectrum was recorded from 550 to 750 nm at 1 nm intervals while fixing the excitation wavelength, which was systematically varied from 530 to 680 nm in 1 nm steps. This measurement scheme resulted in the construction of an EEM, where fluorescence intensities were collected across a two-dimensional grid of excitation and emission wavelengths. These data served as the basis for constructing three-dimensional fluorescence maps and two-dimensional contour plots used in the analysis. Distinct spectral properties of the fluorophores (Supplementary Material Figure S1) allowed for unambiguous identification of ligated products corresponding to each *KRAS* variant, and the simultaneous detection of multiple fluorophores in a single sample further enabled discrimination between homozygous and heterozygous genotypes.

## 3. Results and Discussion

### 3.1. Validation with Synthetic Templates

To validate the sequence-specific ligation fidelity of the bead-coupled LDR assay prior to its application to genomic DNA extracted from cell lines, we first revalidated the fluorophore-specific signatures using synthetic oligonucleotide templates. Although this ligation behavior had already been demonstrated in our previous study [30], representative data are presented here to provide a reference framework for subsequent measurements using genomic DNA.

As summarized in Table 1, each synthetic template was designed as a 43-mer oligonucleotide incorporating a single-nucleotide substitution at position c.35 of the *KRAS* gene (codon 12), thereby mimicking representative variants such as c.35G>A, c.35G>T, and c.35G>C. In each LDR, a mixture of four discriminating primers was included. These primers differ at their 3′-terminal base to match one of the possible variants and are individually labeled at the 5′ end with a distinct fluorophore (HEX, TMR, ROX, or AF647). Although all four primers were present in the reaction mixture, only the discriminating primer with a perfectly matched 3′-terminal base undergoes ligation with the common primer. The resulting LDR product retains the fluorophore of the ligated discriminating primer, which is subsequently detected in the fluorescence emission spectrum.

**Figure 2:**
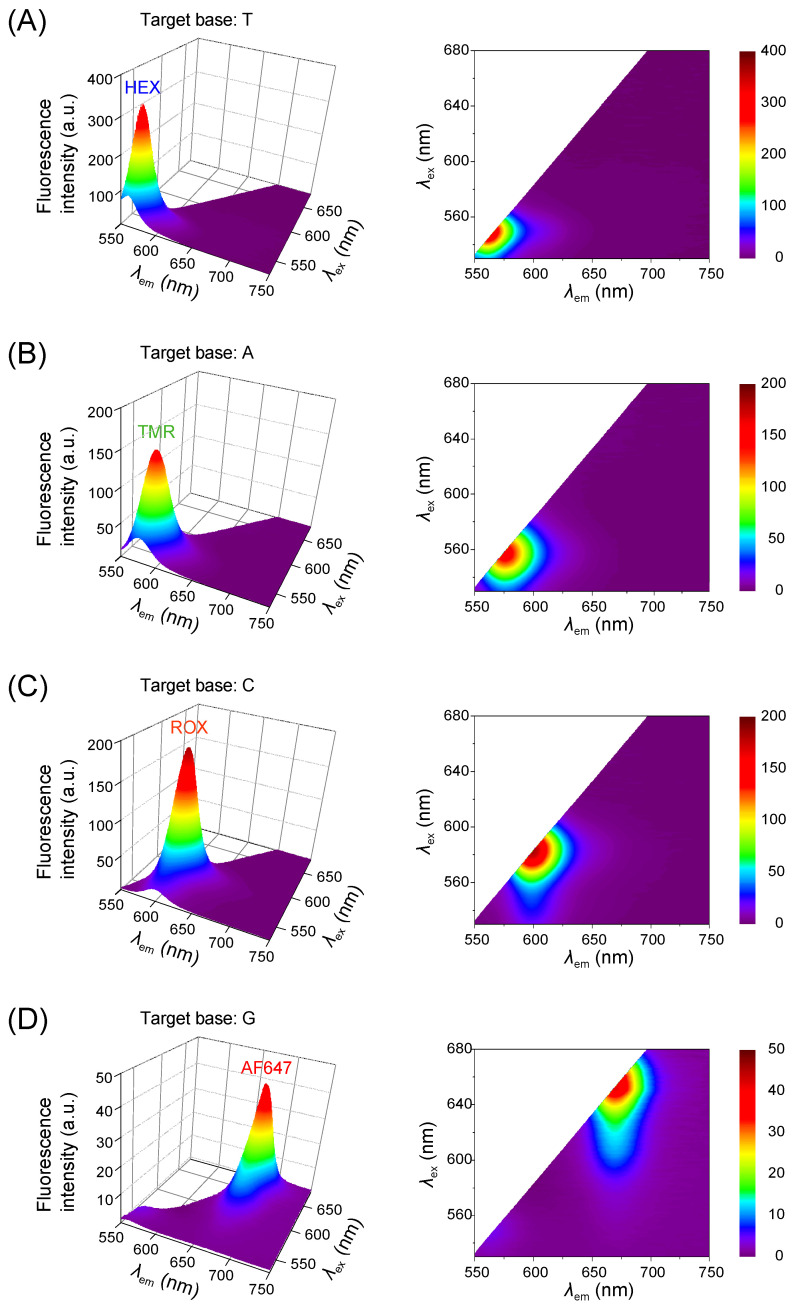
Fluorescence excitation–emission matrix profiles of LDR products generated using synthetic oligonucleotide templates mimicking *KRAS* codon 12 variants. Panels (**A**–**D**) correspond to LDRs using synthetic 43-mer templates designed to contain a single-base substitution at position 35, modeling the c.35 variants observed in *KRAS* codon 12 mutations (see Table 1; the variable bases are shown in bold). Each of panels (**A**–**D**) consists of a left panel displaying a three-dimensional color map of fluorescence intensity and a right panel showing the corresponding two-dimensional contour map. Fluorescence signals were observed only for the fluorophore-labeled discriminating primer whose 3′-terminal base was complementary to the target base in the synthetic template, demonstrating precise base recognition by the LDR assay. The correspondence among each *KRAS* variant, the substituted base incorporated into the synthetic template, and the fluorophore-labeled discriminating primer that recognizes it is summarized in Supplementary Table S1. Abbreviations used are TMR for TAMRA and AF647 for Alexa Fluor 647.

This selective ligation behavior produced fluorescence profiles that unambiguously reflected the nucleotide identity at the target site (Figure 2). For example, in Figure 2A, the synthetic template contained a thymine (T) at position c.35, corresponding to the *KRAS* c.35G>A (p.G12D) mutation. Accordingly, the HEX-labeled discriminating primer with a 3′-terminal adenine (A) was selectively ligated, yielding a fluorescence signature characteristic of HEX. Likewise, the results shown in Figure 2B–D confirmed the expected correspondence for the other nucleotides, namely TMR for T, ROX for G, and AF647 for C, thus validating the assignment across all possible variants. To further assess the practical detection capability of this platform, we examined synthetic oligonucleotide templates at one-tenth of the concentration used in Figure 2 (10 nM → 1 nM). The excitation and emission maxima of each mutant remained identical within ± 2 nm to those observed at higher input levels, while the fluorescence intensity decreased as expected with lower template input (Supplementary Figure S2). These results demonstrate that selective ligation and fluorophore-specific recognition were preserved even at this reduced template level, confirming the reproducibility and stability of the fluorescence readout under the present conditions.

In summary, the assignment used throughout this study is HEX for A, TMR for T, ROX for G, and AF647 for C, as also listed in Table 1. Supplementary Table S1 further clarifies this design by summarizing the relationship among the base at position c.35 in the template, the complementary 3′-terminal base of the discriminating primer, and its fluorescent label. This correspondence, established here with synthetic templates, was consistently applied in subsequent assays using PCR-amplified genomic DNA (as described later in Section 3.3), and its robustness was further examined in the following section.

### 3.2. Spectral Distinctiveness of Fluorophores

**Figure 3:**
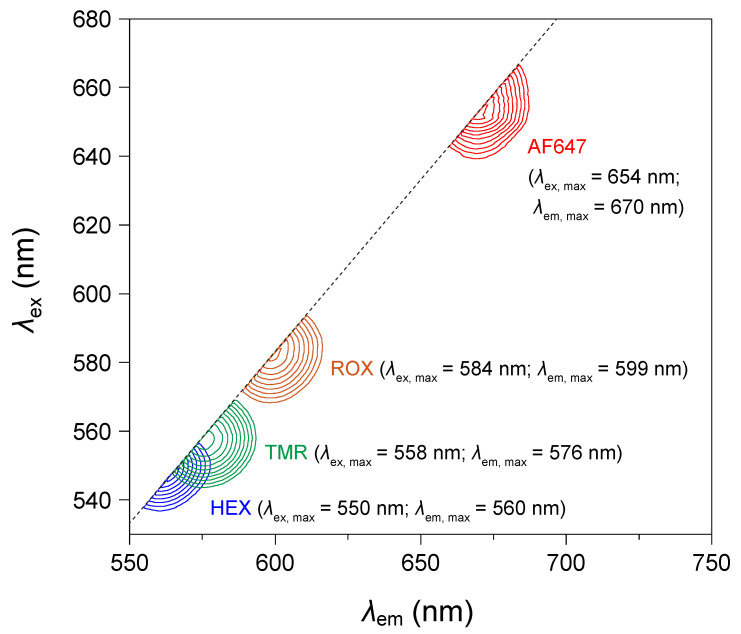
Overlaid contour maps of fluorescence excitation–emission profiles for the four fluorophores used in the LDR assay. To illustrate the spectral distinctiveness among the four fluorophores—HEX, TAMRA (TMR), ROX, and Alexa Fluor 647 (AF647)—used for discriminating *KRAS* codon 12 variants, two-dimensional excitation–emission contour plots derived from the same LDR measurements shown in Figure 2 were overlaid. This composite representation highlights the unique excitation and emission profiles of each fluorophore, supporting their unambiguous discrimination in multiplexed detection assays.

To further substantiate this correspondence between nucleotide identity and fluorophore signals, we overlaid the contour maps of the four fluorophores used (HEX, TMR, ROX, and AF647) in a composite plot (Figure 3) to better visualize their spectral distinctiveness. Each fluorophore exhibits a unique excitation–emission profile, as shown in the two-dimensional contour maps derived from the EEM data in Figure 2. This composite representation highlights the non-overlapping fluorescence signatures among the four dyes, allowing for their unambiguous discrimination within the same assay. Such spectral separation is essential for the simultaneous discrimination of different *KRAS* genotypes at codon 12, ensuring accurate interpretation of LDR products in both homozygous and heterozygous samples.

### 3.3. Application to Genomic DNA and Signal Considerations

Having validated the ligation specificity and fluorophore-based discrimination using synthetic oligonucleotide templates, we next applied the LDR assay to PCR-amplified genomic DNA extracted from colorectal cancer cell lines with known *KRAS* codon 12 genotypes (Table 2). LDR requires a single-stranded template, and since PCR products are double-stranded, only one strand can serve as the effective template. In our assay design, for the LDR template we consistently used the strand of the PCR product complementary to the discriminating primers. For example, while the wild-type base at c.35 is G, a c.35G>A mutation necessarily results in a T on the opposite strand. By selecting this T-containing strand as the LDR template, only the discriminating primer with a 3′-terminal A can ligate, and thus the 3′-terminal base directly represents the reported allele at position c.35. As noted in Section 3.1, the correspondence between each allele and its fluorophore-labeled discriminating primer was defined as HEX for A, TMR for T, ROX for G, and AF647 for C (Table 1), and the underlying relationship among the base at position c.35 in the LDR template, the 3′-terminal base of the discriminating primer, and its fluorescent label is summarized in Supplementary Table S1. This mapping enabled us to assign each observed fluorescence signal directly to the nucleotide identity at c.35 in the PCR-amplified genomic DNA.

**Figure 4:**
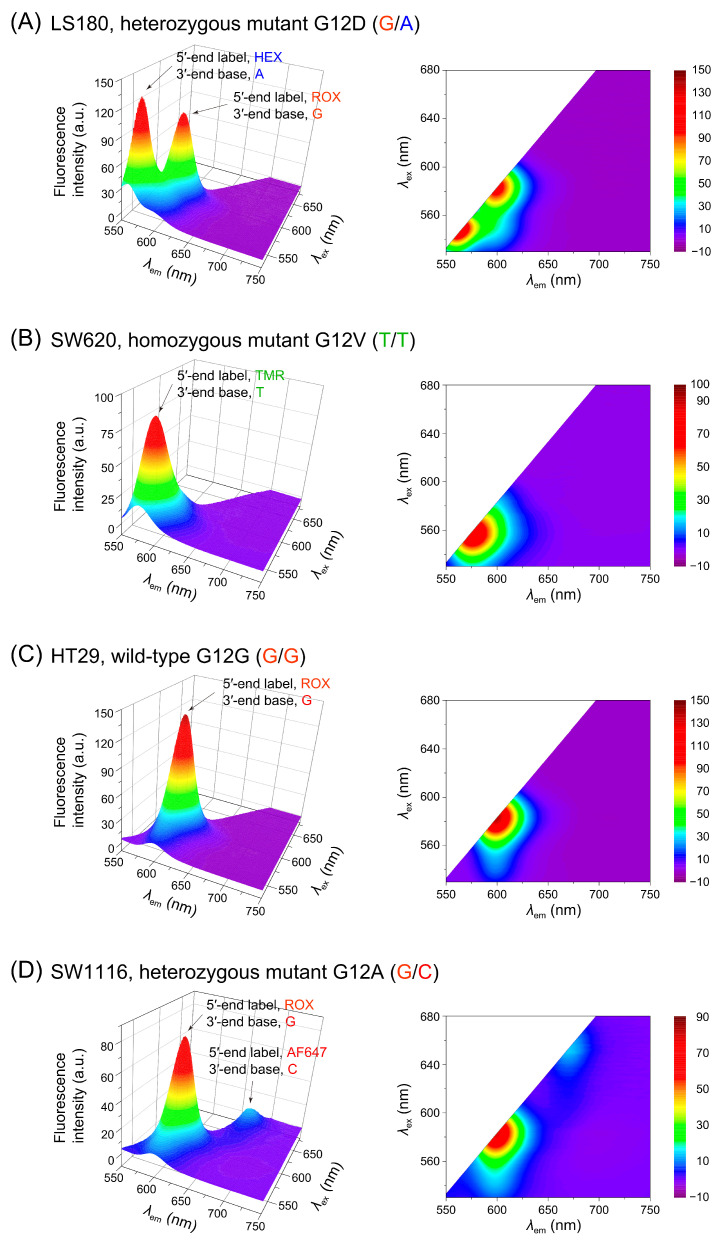
Fluorescence excitation–emission matrix contour profiles of LDR products derived from PCR-amplified genomic DNA of four colorectal cancer cell lines with different *KRAS* codon 12 genotypes. Panels (**A**–**D**) correspond to LS180 (G/A), SW620 (T/T), HT29 (G/G), and SW1116 (G/C), respectively. Each contour map shows the EEM profile of purified LDR products generated by selective ligation with fluorophore-labeled discriminating primers. Fluorescence signals appeared only for the primer(s) whose 3′-terminal base matched the target base at position c.35, enabling identification of each genotype. Dual signals in panels (**A**,**D**) indicate heterozygous genotypes (G/A and G/C), whereas single-signal profiles in panels (**B**,**C**) correspond to a homozygous mutant (T/T) and the wild-type allele (G/G), respectively. In each panel, the annotation (e.g., “5′-terminal label, ROX; 3′-terminal base, G”) denotes the discriminating primer that responded to the template: the 5′ end indicates the attached fluorophore, while the 3′ end specifies the base complementary to position c.35. Because the assay was designed to use the complementary strand as the template, the 3′-terminal base of the discriminating primer directly represents the allele composition at this position. Abbreviations used are TMR for TAMRA and AF647 for Alexa Fluor 647.

For instance, the LS180 cell line carries the heterozygous G12D mutation (G/A), meaning that its genomic DNA contains both G and A at position c.35 (Table 2). According to the template design, this results in complementary C and T bases on the LDR template strand. Consequently, both the ROX-labeled discriminating primer with a 3′-terminal G and the HEX-labeled primer with a 3′-terminal A were selectively ligated. The EEM profile of this sample (Figure 4A) clearly exhibits distinct spectral signatures from ROX and HEX, confirming the presence of both alleles and validating the genotype as G/A. Similar logic applies to the other cell lines: SW620, which harbors a homozygous G12V mutation (T/T), yielded a single TMR signal (Figure 4B); HT29 (G/G) produced only a ROX signal corresponding to the wild-type allele (Figure 4C); and SW1116, carrying the heterozygous G12A mutation (G/C), exhibited dual signals from ROX and AF647 (Figure 4D). These results demonstrate that the assay reliably distinguishes homozygous and heterozygous *KRAS* genotypes in genomic DNA based on distinct fluorophore-specific spectral patterns. It should also be noted that no additional fluorescence peaks attributable to mismatched discriminating primers were observed, supporting high ligation specificity under the present conditions; at substantially lower template concentrations, minor cross-ligation cannot be excluded in principle and will be investigated in future optimization studies.

Although the emission spectra of HEX and TMR partially overlap (Figure 3), this overlap does not interfere with accurate genotype identification in the present study because each *KRAS* codon 12 mutation corresponds to a single-nucleotide substitution producing one fluorophore-specific signature. The combination of HEX (A) and TMR (T) signals would occur only in a hypothetical heterozygous G12D/G12V configuration, which has not been reported in colorectal cancer. If such a case were to be analyzed, alternative fluorophore pairs with non-overlapping spectra could be selected without altering the assay principle.

Notably, the AF647 signal—assigned to cytosine (c.35C)—appeared weaker than the signals of the other fluorophores, even for heterozygous samples where the two alleles are expected to be present at comparable proportions after PCR. This trend was also observed in measurements using synthetic templates under identical concentrations (Figure 2), indicating that the difference is not attributable to allelic imbalance but rather to the detection pathway. A plausible explanation is either (i) the reduced sensitivity of the spectrofluorometer in the far-red region, (ii) the lower fluorescence quantum yield of AF647 relative to the other dyes, or a combination of both. Because all spectra were acquired on the same FP-6500 instrument using identical settings, the weaker apparent brightness of AF647 can be attributed to these instrumental and dye-related factors, rather than to measurement conditions. Importantly, the fluorophore–base mapping established with synthetic templates (HEX→A, TMR→T, ROX→G, AF647→C; Table S1) held consistently in genomic DNA analysis, so genotype calls were unaffected. As a practical improvement, replacing AF647 with a spectrally compatible but brighter far-red dye (maintaining minimal spectral overlap with HEX, TMR, and ROX) would be expected to increase the cytosine-assigned signal without altering assay logic.

## 4. Conclusions

In this study, we extended our bead-coupled LDR assay from synthetic oligonucleotides to genomic DNA extracted from colorectal cancer cell lines, enabling reliable identification of clinically relevant *KRAS* codon 12 mutations. The assay successfully distinguished both homozygous and heterozygous genotypes through fluorophore-specific EEM signatures. This single-tube workflow, which integrates magnetic bead capture and fluorescence-based readout, provides a rapid, accessible, and cost-effective approach compared with conventional methods such as electrophoresis or microarray hybridization.

Among these, electrophoresis has historically been the most common technique for separating single-stranded LDR products from unreacted fluorescent primers, but it requires polyacrylamide gels, lengthy run times, and dedicated fluorescence-imaging equipment. Capillary electrophoresis can achieve higher resolution but depends on costly automated instruments (e.g., Applied Biosystems Genetic Analyzer [10,11,14,15,16,17,19,20]). In contrast, the present bead-coupled LDR eliminates any electrophoretic or hybridization step, and the resulting eluates are directly analyzed using a standard spectrofluorometer available in most molecular laboratories, thereby reducing overall operation time and running cost.

Although the AF647 signal appeared consistently weaker than those of the other fluorophores, this effect reflected instrumental and dye-specific properties rather than allelic imbalance, and genotype calls remained unaffected. Practical improvements, such as substituting AF647 with a brighter far-red dye, could further enhance detection sensitivity without altering the assay principle. Overall, our results demonstrate the robustness and practicality of the LDR–EEM platform for single-nucleotide variation analysis, highlighting its potential utility not only in research but also in future diagnostic applications.

Beyond the *KRAS* model, the same bead-coupled LDR–EEM workflow can be adapted to other clinically relevant point mutations by redesigning the allele-discriminating primers—for example, *NRAS* codon 61 or *BRAF* V600E. In subsequent studies, we plan to assess the method using DNA isolated from clinical biopsy specimens to evaluate robustness in the presence of sample heterogeneity and potential PCR inhibitors. These directions align with the platform’s emphasis on simple sample handling and readout on a standard spectrofluorometer, and they outline a practical path toward broader diagnostic utility.

## Figures and Tables

**Table 1:** Oligonucleotide sequences used for target amplification by PCR and subsequent LDR detection of *KRAS* mutations.

Primer Name	Usage	Sequence (5′→3′)	Size (mer)
*KRAS exon 2* forward	Primer for PCR	TTAAAAGGTACTGGTGGAGTATTTGATA	28
*KRAS* exon 2 reverse	Primer for PCR	AAAATGGTCAGAGAAACCTTTATCTGT	27
*KRAS* c.35A-HEX	Discrim. primer for LDR	HEX-AAACTTGTGGTAGTTGGAGCTGA	23
*KRAS* c.35T-TAMRA	Discrim. primer for LDR	TAMRA-AAACTTGTGGTAGTTGGAGCTGT	23
*KRAS* c.35G(WT)-ROX	Discrim. primer for LDR	ROX-AAACTTGTGGTAGTTGGAGCTGG	23
*KRAS* c.35C-Alexa Fluor 647	Discrim. primer for LDR	Alexa Fluor 647-AAACTTGTGGTAGTTGGAGCTGC	23
*KRAS* c.36T Com-2/3′-biotin	Com. primer for LDR	*p*TGGCGTAGGCAAGAGTGCCT-biotin	20
LDR template for p.G12D	Synthetic template for LDR	AGGCACTCTTGCCTACGCCA**T**CAGCTCCAACTACCACAAGTTT	43
LDR template for p.G12V	Synthetic template for LDR	AGGCACTCTTGCCTACGCCA**A**CAGCTCCAACTACCACAAGTTT	43
LDR template for p.G12G(WT)	Synthetic template for LDR	AGGCACTCTTGCCTACGCCA**C**CAGCTCCAACTACCACAAGTTT	43
LDR template for p.G12A	Synthetic template for LDR	AGGCACTCTTGCCTACGCCA**G**CAGCTCCAACTACCACAAGTTT	43

Abbreviations: Com., common; Discrim., discriminating; *p*, phosphorylated. HEX, ROX, TAMRA, and Alexa Fluor 647 are fluorophores with distinct spectral properties. Underlined nucleotides indicate the discriminating bases at the 3′-end of LDR primers. Bold letters in synthetic template sequences indicate the target nucleotides corresponding to *KRAS* codon 12.

**Table 2:** *KRAS* codon 12 mutation status in different colorectal cancer cell lines.

Cell Line	Nucleotide Change	Amino Acid Change	Zygosity	Genotype
LS180	c.35G>A	p.G12D	Heterozygous	G/A
SW620	c.35G>T	p.G12V	Homozygous	T/T
HT-29	c.35G (WT)	p.G12G	Homozygous	G/G
SW1116	c.35G>C	p.G12A	Heterozygous	G/C

## Data Availability

Data is contained within the article or Supplementary Material.

## Supplementary Materials

The following supporting information can be downloaded at: https://www.mdpi.com/article/10.3390/mps8060142/s1, Figure S1: Spectral properties of the fluorophores used in the LDR assay; Figure S2: Fluorescence excitation–emission profiles of LDR products generated using 1 nM synthetic oligonucleotide templates corresponding to *KRAS* codon 12 variants; Table S1: Relationship between *KRAS* codon 12 variant, target base, and fluorophore-labeled discriminating primer.
