# Supplementary material for: Fluorescence-Based Detection of KRAS Mutations in Genomic DNA Using Magnetic Bead-Coupled LDR Assay
Source: Methods Protoc. 2025 Dec 1;8(6):142. doi: 10.3390/mps8060142 (PMC12736157; doi:10.3390/mps8060142)
Supplement: Supplementary file 1 [file mps-08-00142-s001.zip › mps-3926477-supplementary.pdf]

Supplementary Information

Fluorescence-Based Detection of *KRAS* Mutations in Genomic DNA Using Magnetic Bead-Coupled LDR Assay

Chika Morimoto [1] and Masahiko Hashimoto [1,*]

[1]Department of Chemical Engineering and Materials Science, Faculty of Science and Engineering, Doshisha University, 1-3 Tataramiyakodani, Kyotanabe, Kyoto 610-0321, Japan

***Correspondence:** Prof. Masahiko Hashimoto, Department of Chemical Engineering and Materials Science, Faculty of Science and Engineering, Doshisha University, 1-3 Tataramiyakodani, Kyotanabe, Kyoto 610-0321, Japan
E-mail: mahashim@mail.doshisha.ac.jp
Fax: +81-774-65-6594

Table of Contents

1. Figure S1 **Spectral properties of the fluorophores used in the LDR assay**

2. Figure S2 **Fluorescence excitation–emission profiles of LDR products generated using 1 nM synthetic oligonucleotide templates corresponding to *KRAS* codon 12 variants**

3. Table S1 **Relationship between *KRAS* codon 12 variant, target base, and fluorophore-labeled discriminating primer**

1. Figure S1 Spectral properties of the fluorophores used in the LDR assay

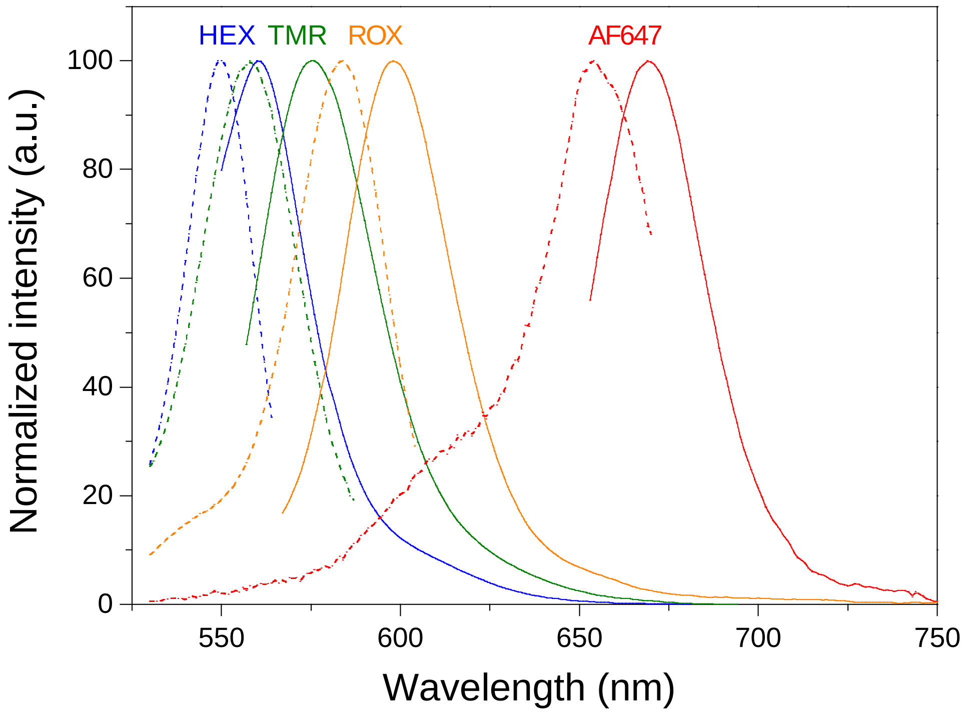

Excitation (dashed lines) and emission (solid lines) spectra of the four fluorophores used for labeling discriminating primers in the LDR assay: HEX (blue), TAMRA (green), ROX (orange), and Alexa Fluor® 647 (red). For clarity, TAMRA and Alexa Fluor® 647 are abbreviated as TMR and AF647, respectively, in the figure. The distinct spectral profiles of each fluorophore enable unambiguous discrimination of ligated products based on their fluorescence characteristics. Spectra were normalized and stacked for visualization.

2. Figure S2 Fluorescence excitation–emission profiles of LDR products generated using 1 nM synthetic oligonucleotide templates corresponding to *KRAS* codon 12 variants

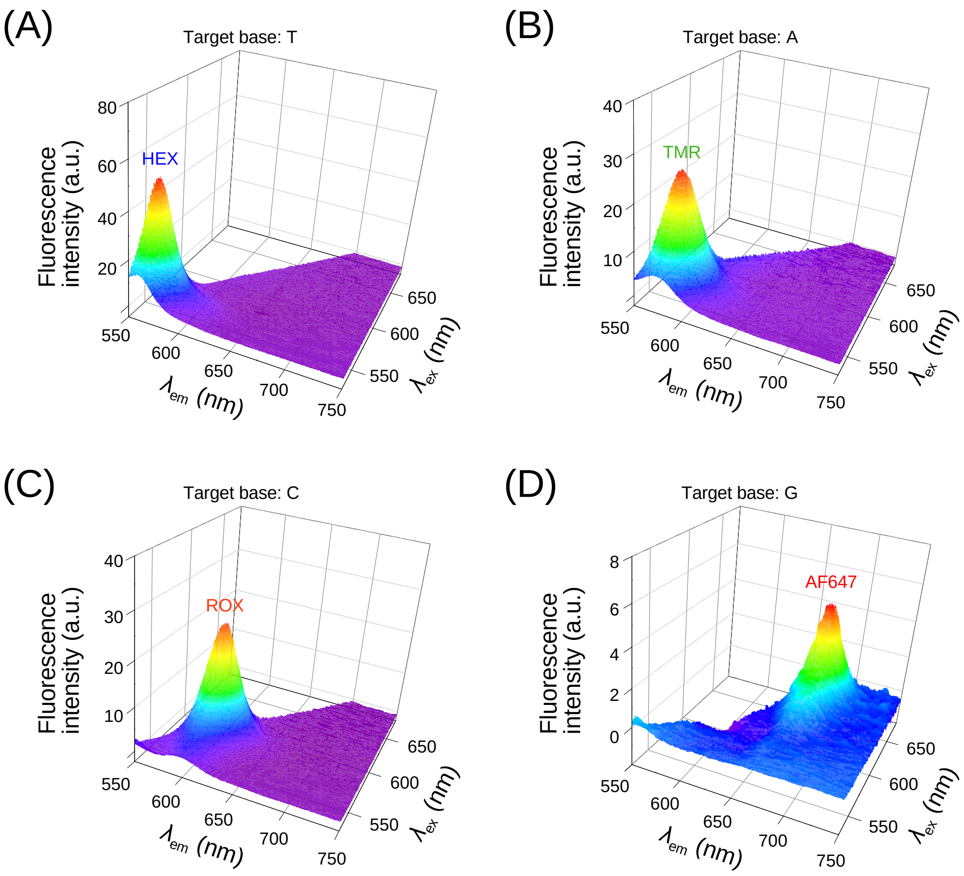

Experimental conditions were identical to those described for Figure 2 in the main text, except that the input template concentration was reduced to one-tenth (1 nM). Panels (A–D) correspond to templates containing the target bases T, A, C, and G, respectively, each producing fluorescence signals from the discriminating primer labeled with the corresponding fluorophore: HEX for T, TMR for A, ROX for C, and AF647 for G. The characteristic excitation and emission maxima remained unchanged, confirming that selective ligation and fluorophore-specific recognition were preserved even at the lower template concentration.

2. Table S1 Relationship between *KRAS* codon 12 variant, target base, and fluorophore-labeled discriminating primer

KRAS variant	Base at position c.35 in the LDR template	3′-terminal base of discriminating primer	Fluorophore label
p.G12D	T	A	HEX
p.G12V	A	T	TAMRA (TMR)
p.G12G (WT)	C	G	ROX
p.G12A	G	C	Alexa Fluor® 647 (AF647)

This table summarizes the base-pairing relationship between each *KRAS* codon 12 variant and the corresponding fluorophore-labeled discriminating primer used in the LDR assay. For each variant, the target base at position c.35 determines the 3′-terminal base of the complementary discriminating primer, which is labeled at the 5′ end with a specific fluorophore. This correspondence allows each variant to be identified by its characteristic fluorescence signature, as demonstrated in Figure 2.
